# Zur Schnittmenge von Informatik mit Friedens- und Sicherheitsforschung: Erfahrungen aus der interdisziplinären Lehre in der Friedensinformatik

**DOI:** 10.1007/s42597-022-00078-4

**Published:** 2022-09-20

**Authors:** Christian Reuter, Thea Riebe, Jasmin Haunschild, Thomas Reinhold, Stefka Schmid

**Affiliations:** Wissenschaft und Technik für Frieden und Sicherheit (PEASEC), Technische Universität Darmstadt, Darmstadt, Deutschland

**Keywords:** Informatik, Cyberkrieg, Cyberfrieden, Interdisziplinarität, Lehre, Computer science, Cyber war, Cyber peace, Interdisciplinarity, Teaching

## Abstract

Interdisziplinäre Forschung und Lehre zwischen Informatik sowie Friedens- und Sicherheitsforschung ist vor dem Hintergrund der Bedeutung möglicher Konflikte im Cyberspace unabdingbar. Auch wenn sowohl in der einen als auch der anderen Disziplin zahlreiche etablierte Lehrveranstaltungen und Lehrbücher existieren, gilt dies nicht für deren Schnittmenge. Dieser Beitrag reflektiert die Einführung der in Bezug auf Thematik und Hörerschaft interdisziplinären Lehrveranstaltung „Informationstechnologie für Frieden und Sicherheit“ für Studierende der Informatik, IT-Sicherheit und Wirtschaftsinformatik der Technischen Universität Darmstadt sowie Friedens- und Konfliktforschung der TU Darmstadt in Kooperation mit der Goethe-Universität Frankfurt. Hierbei werden Herausforderungen und Lösungsansätze der interdisziplinären Lehre dargestellt und die Bedeutung dieser Lehre hervorgehoben.

## Abstract (en)

Interdisciplinary research and teaching between computer science and peace and security studies is indispensable against the background of the significance of possible conflicts in cyberspace. Even though numerous established courses and textbooks exist in both the one and the other discipline, this does not apply to their intersection. This article reflects on the introduction of the interdisciplinary course “Information Technology for Peace and Security” for students of Computer Science, Cyber Security and Information Systems at Technical University of Darmstadt and Peace and Conflict Research at the TU Darmstadt in cooperation with the Goethe University Frankfurt. The challenges and solutions of interdisciplinary teaching are presented and the importance of this teaching is emphasised

## Einleitung: Interdisziplinäre Forschung und Lehre zwischen Technik und Frieden

Politische Konflikte, die im Cyberspace ausgetragen werden, gewinnen zunehmend an Bedeutung und stellen dabei ein komplexes empirisches Problem für die Friedens- und Konfliktforschung dar. Die ersten Cyberattacken im Kontext von bewaffneten Konflikten fanden bereits vor etwa 15 Jahren statt (Reinhold und Reuter 2019): 2007 soll das israelische Militär die syrischen Luftabwehrsysteme sabotiert haben, und in Estland wurden Server vermutlich durch kremlnahe Aktivisten von Russland aus angegriffen und zeitweise lahmgelegt. Im Georgien-Krieg 2008, sowie 2014 bei der Annexion der Krim wurden gezielte Hacking-Attacken festgestellt, ebenso wie DDoS-Attacken, die die Verfügbarkeit von Internetdiensten durch gezielte Überlastungen (z. B. durch Anfragen mit Bots) beeinträchtigen. Auch deutsche Regierungssysteme wurden bereits 2015 und 2017 Opfer gezielter, mutmaßlich staatlich gesteuerter Cyberattacken. Vor allem jedoch der russische Überfall auf die Ukraine im Jahr 2022 verdeutlicht, dass Cyberattacken zunehmend als Vorbereitung auf physische Angriffe und als Störung des Gegners als Teil einer hybriden Kriegsführung eingesetzt werden. Dabei ist auch von einem Effekt auf die internationale Kooperation auszugehen, da andere staatliche Akteure Cyberattacken zunehmend wie physische Kriegshandlungen werten (vgl. z. B. The White House 2022).

Die vielfältigen Arten, auf die digitale Technologien genutzt werden, um neue (digitale) militärische Angriffe, häufig aber auch um alte Strategien mit neuen Mittel zu unterstützen, wurden bisher allerdings entweder in der Friedens- und Konfliktforschung oder in der Informatik diskutiert. Aufbauend auf disziplinären Perspektiven blieb die Forschung in ihren Analysen größtenteils selektiv. So wurde einerseits vor allem über Rüstungskontrolle und das Konzept des *Cyber Wars* diskutiert (Werkner und Schörnig 2019), sowie andererseits an einer Einordnung unterschiedlicher Attacken und forensischer Möglichkeiten der Attribution gearbeitet (Nisioti et al. 2018). Zur Stärkung einer interdisziplinären Perspektive, welche friedenspolitische wie technische Aspekte gebündelt betrachtet, fokussiert sich die naturwissenschaftlich-technische Friedens- und Konfliktforschung (Altmann et al. 2017) auch auf Konfliktdynamiken und Kooperationspotenziale vor allem mit Blick auf staatlich-geförderte Aktivitäten im Cyberspace. Durch interdisziplinäre Zusammenarbeit kann so wertvolles Wissen über Bedrohungsszenarien und technologische Möglichkeiten in immer drängender werdende Friedensbestrebungen integriert werden.

Ebenso findet Lehre regelmäßig in disziplinären Silos statt, weshalb eine einführende und für Studierende verschiedener Disziplinen gleichermaßen verständliche Zusammenstellung relevanter Konzepte und Thematiken einen wichtigen Beitrag leisten kann. Wir betrachten im Folgenden die Lehrveranstaltung „Informationstechnologie für Frieden und Sicherheit“ als Beispiel für eine in Bezug auf Thematik und Hörer*innenschaft interdisziplinäre Lehrveranstaltung. Dies erlaubt die Herausstellung von Potenzialen, die es weiter auszuschöpfen gilt, lenkt den Blick allerdings auch auf Limitationen, die eine praktische Umsetzung interdisziplinären Austauschs mit sich bringt. Sie wird seit dem Wintersemester 2018/2019 als integrierte Lehrveranstaltung mit Vorlesungs- und Übungsanteilen im Umfang von vier Semesterwochenstunden jedes zweite Semester angeboten. Die thematische Auseinandersetzung mit Cyberkrieg, -konflikten, und -frieden aus einer interdisziplinären Perspektive ist in der Lehre noch wenig etabliert und ermöglicht den involvierten Studierenden den Zugang zu sich gegenseitig ergänzendem Wissen.

Zwar existieren bereits zahlreiche etablierte Lehrbücher der Friedens- und Konfliktforschung (Gießmann und Rinke 2019; Imbusch und Zoll 2010; Schlotter und Wisotzki 2011; Werkner 2020) oder der Informatik mit ihren dazugehörigen breitgefächerten Teilbereichen Cybersicherheit (Rashid et al. 2021), Mensch-Computer-Interaktion (Dix et al. 2013) oder Informatik und Gesellschaft (Quinn 2018). Allerdings existieren nur wenige Publikationen, die die Schnittmenge von Informatik und Friedens- und Sicherheitsforschung adressieren. Dies haben wir als Desiderat wahrgenommen, vor allem vor dem Hintergrund der Bedeutung der gesamten naturwissenschaftlich-technischen Friedensforschung (Altmann et al. 2017), die deutschlandweit prekärerweise mittlerweile nur noch mit sehr wenigen Professuren vertreten ist (Reuter et al. 2020), als auch mit Blick auf die Bedeutung der „Friedensinformatik“ als Fachgebiet.

Die interdisziplinäre Ausrichtung, welche sich in der Forschungsliteratur sowie angebotenen Lehrveranstaltungen niederschlägt, wurde 2017 mit dem an der Technischen Universität Darmstadt eingerichteten Lehrstuhl Wissenschaft und Technik für Frieden und Sicherheit (engl. Science and Technology for **Pea**ce and **Sec**urity, PEASEC) institutionalisiert. Hier wird Informatik mit Friedens- und Sicherheitsforschung im Fachbereich Informatik mit Zweitmitgliedschaft im Fachbereich Gesellschafts- und Geschichtswissenschaften verbunden. In der Schnittmenge der Disziplinen Cyber-Sicherheit und -Privatheit, Friedens- und Konfliktforschung sowie Mensch-Computer-Interaktion adressiert PEASEC grundlegende Fragen zu Frieden und Krieg im Cyberspace und zur Rüstungskontrolle (Reinhold und Reuter 2021), zur Dual-Use-Problematik in der Informatik (Riebe et al. 2021), sowie zu friedens- und sicherheitsfördernden als auch konfliktiven Interaktionen mit und in sozialen Medien (Reuter und Kaufhold 2018). Diese Themen lassen sich durch das Lehr-Engagements des Lehrstuhls sowohl im Fachbereich der Informatik, insbesondere in den Bachelor- und Masterstudiengängen Informatik, IT-Sicherheit und Wirtschaftsinformatik (jeweils integriert als Wahlpflichtmodule), sowie im Fachbereich der Gesellschafts- und Geschichtswissenschaften, und hier insbesondere im Masterstudiengang Internationale Studien/Friedens- und Konfliktforschung, verbinden. Generell steht ein Besuch der Lehrveranstaltung allen Interessierten offen, die den interdisziplinären Studienschwerpunkt „Wissenschafts- und Technikforschung“ an der TU Darmstadt wählen. Dieser Beitrag stellt die Veranstaltung vor und diskutiert die Herausforderungen einer interdisziplinären Veranstaltung mit sehr diverser Hörer*innenschaft, sowie *Best Practice*-Erfahrungen aus dem Lehrbetrieb.

## Die Lehrveranstaltung: Informationstechnologie für Frieden und Sicherheit

Unsere Erfahrungen mit der Lehrveranstaltung „Informationstechnologie für Frieden und Sicherheit“ in den vier Wintersemestern von 2018/2019 bis 2021/2022 werden im Folgenden zunächst mit der Darstellung des Konzepts der im wöchentlichen Wechsel im Umfang von etwa drei Zeitstunden angebotenen Vorlesung und Übung, dem digitalen Lehrbetrieb während COVID-19 sowie auf der Ergebnisse und Evaluationen der Lehrveranstaltungen reflektiert, bevor Herausforderungen und Kernbeobachtungen diskutiert werden.

### Lehrkonzept: Aufbereitung und Vermittlung von Wissen

Basierend auf den Erfahrungen aus unserem 2018 herausgegebenen Lehrbuch *Sicherheitskritische Mensch-Computer-Interaktion: Interaktive Technologien und Soziale Medien im Krisen- und Sicherheitsmanagement* (Reuter 2018), wurde 2019 das Lehrbuch *Information Technology for Peace and Security – IT-Applications and Infrastructures in Conflicts, Crises, War, and Peace* (Reuter 2019) als grundlegende Einführung in die Grenzbereiche der Informatik und Friedens- und Konfliktforschung umgesetzt. Dabei werden Konflikte, Krieg und Frieden im Cyberraum, Cyber-Rüstungskontrolle, Cyber-Attribution und -Infrastrukturen sowie Kultur und Interaktion näher beleuchtet, bevor abschließend ein Ausblick gegeben wird. Die einführende Lehrveranstaltung orientiert sich in ihrer Struktur stark am Aufbau des Buches. Durch die Beteiligung von Autor*innen verschiedenster Disziplinen (z. B. Sicherheitspolitik oder Cybersecurity), sowie die anschließende Reflexion der vorgestellten Beiträge wurde eine inhärent interdisziplinäre Grundlage geschaffen. Beginnend mit der Einführung abstrakterer politikwissenschaftlicher Grundbegriffe und theoretischer Konzepte (z. B. Krieg, Konflikt, Sicherheitsdilemma) erlaubt das Lehrbuch im schrittweisen Durchgang eine weitere Konkretisierung konfliktiver und kooperativer Szenarien durch die Illustrierung sozio-technischer Problematiken und Möglichkeiten. Dies erlaubt auch das Schlagen einer epistemologischen Brücke, zwischen dem wissenschaftlichen Anspruch etwas besser zu verstehen (z. B. Welche verschiedenen Dimensionen von Gewalt können vorherrschen?) sowie problemlösungsorientiert (z. B. Wie können systematisch Abwehrmechanismen gegen Cyberangriffe entwickelt werden?) zu denken. Dabei werden diese Anliegen nicht immer strikt getrennt voneinander angegangen. So werden Rüstungskontrollmaßnahmen inklusive der Entwicklung technischer Hilfsmittel im Kontext einer Analyse staatlichen Verhaltens in den internationalen Beziehungen diskutiert. Zudem bietet die Interpretation des Darknets als zu versicherheitlichendes Problem für Informatiker*innen die Möglichkeit, verschiedene Bestrebungen der Attribution (und Strafverfolgung) im politischen Kontext zu begreifen.

Während für Studierende der Sozialwissenschaften der Umgang mit jenen Grundbegriffen tendenziell vertraut ist, hilft technisches Vorwissen den Informatikstudierenden beim Transfer friedenspolitischer Fragen auf empirische Fälle der Nutzung von IT im Kontext von Frieden und Sicherheit. Abschließende Fragen der jeweiligen Kapitel erlauben an geeigneten Stellen die Rekapitulation des neuerworbenen Wissens. An diesem iterativen Vorgehen, welches die (Selbst‑)Prüfung des Erlernten in den Vordergrund rückt, orientieren sich die Vorlesung und Übung ebenfalls. Des Weiteren wurden, zur Ebnung eines gemeinsamen Lernraums, Praktiken aus den jeweiligen Disziplinkulturen übernommen, sodass für die Studierendengruppen bekannte Verfahren einen Referenzrahmen im Zuge der Vermittlung unbekannter Themenkomplexe bieten. So ist die Orientierung am Lehrbuch den Studierenden aus der Friedens- und Konfliktforschung vertraut, während die vorausgesetzte Grundlagenlektüre für Informatikstudierende oft die Aneignung einer neuen Routine bedeutet. Ebenso ist die anwendungsorientierte Ausrichtung der Veranstaltung, welche eine Übung mit Fokus auf konkrete Fallbeispiele soziotechnischer Interaktion beinhaltet, für Studierende der Sozialwissenschaften weitgehend unbekanntes Feld.

### Vorlesung zur Vermittlung und Diskussion der Themen

Die *Vorlesung*, die im wöchentlichen Wechsel mit der Übung angeboten wurde, ist dabei in sieben Teile gegliedert. In Teil I: *Einleitung und Grundlagen *erfolgt eine Einführung in die naturwissenschaftlich-technische Friedensforschung und insbesondere IT in Friedens‑, Konflikt- und Sicherheitsforschung. Teil II behandelt *Cyber-Konflikte und -Krieg* mit seinen Bestandteilen Informationskrieg, Cyberspionage und Cyberattacken sowie Darknets als Instrument zur Cyber-Kriegsführung. Teil III, *Cyber-Frieden* soll Wege vom Cyber-Krieg zum Cyber-Frieden aufzeigen, Dual-Use und Dilemmata in der Cyber-Sicherheit, sowie Vertrauens- und sicherheitsbildende Maßnahmen besprechen. Teil IV, *Cyber-Rüstungskontrolle* behandelt die Rüstungskontrolle, ihre Anwendbarkeit und neue Konzepte für den Cyberwaffen sowie unbemannte Systeme und die Cyber-Verifikation. Teil V: *Cyber-Attribution und -Infrastrukturen* befasst sich mit Attribution von Cyberattacken sowie resilienten und sicheren kritischen Infrastrukturen. Teil VI: *Soziale Interaktion* adressiert die Aufteilung von Safety und Security, kulturelle Gewalt sowie die Nutzung von sozialen Medien und Informations- und Kommunikationstechnologie in Krisengebieten. Teil VII: *Ausblick* wagt eine Prognose zur Zukunft von IT in Frieden und Sicherheit. Die von 50 bis 150 Studierenden besuchte Vorlesung sah die Vermittlung von am Lehrbuch-orientierten Inhalten, jedoch häufig auch die aktive Diskussion mit Studierenden vor. Während in den Präsenzsemestern die verbale Diskussion hervorragend funktionierte, und je nach Fragestellung entweder Informatik-Studierende (etwas mehr zu technischen Aspekten) oder Friedens- und Konfliktforschungsstudierende (zu sicherheitspolitischen Aspekten) wertvolle Beiträge leisten konnten, hat sich dies in der Pandemie etwas verändert. In der Live-Onlinevorlesung wurde eine größere Zurückhaltung zur verbalen Diskussion deutlich, sodass regelmäßige Quizze oder offene Fragen zur Beantwortung im Chat die Diskussion ergänzt haben.

Videoaufzeichnungen der Live-Veranstaltungen werden im Moodle-Kurs gebündelt mit Lernmaterialien aus Übungen sowie externen Veranstaltungen zur Verfügung gestellt sowie die Kommunikation zu den Studierenden kanalisiert. Zudem werden zusätzliche (nicht klausurrelevante) Materialien über die universitäre E‑Learning-Plattform zur Verfügung gestellt. Dabei handelt es sich unter anderem um Vorträge oder Interviews, in denen Expert*innen ihre Forschung praxisnah besprechen. Die Studierenden haben hierbei die Möglichkeit auf relevante Informationen zuzugreifen und weiterführende Thematiken zu erkunden, was den explorativen Charakter der interdisziplinären Debatte betont. Die starke Aktualität der Thematik um IT im Kontext von Krieg und Frieden kam zudem in spontanen Zusatzveranstaltungen zur russischen Invasion in die Ukraine zum Tragen. Hier wurden offene, außerordentliche Informations- und Diskussionsveranstaltungen über Zoom angeboten, die es Anwesenden erlaubten, über relevante Entwicklungen zu sprechen und ihre Rolle als (angehende) Friedens- und Konfliktforscher*in oder Informatiker*in zu reflektieren.

### Übung zur Anwendung, Gruppenarbeit und Präsentation der Inhalte

Schwerpunkt der *Übung* waren die Anwendung und Diskussion der Inhalte mit Blick auf empirische Fälle und Gehalt verschiedener Konzepte. Wie in der Informatik üblich werden zu zahlreichen Vorlesungen begleitende Übungen angeboten. Hierzu wurden, zusätzlich zu den rekapitulierenden Fragen des Lehrbuchs, Fragen entwickelt, deren Beantwortung von den Studierenden vorbereitet und in der Übung präsentiert wurden. Diese sind dazu konzipiert, aktuelle Debatten aufzugreifen, wichtige Organisationen der technischen Friedens- und Konfliktforschung kennenzulernen und bedeutende historische Fälle aufzugreifen. Aufgaben waren beispielsweise: „Was versteht man unter der Militarisierung des Cyberspace und welche gesellschaftlichen und internationalen Risiken entstehen dadurch? Erläutern Sie anhand eines realen Beispiels“ oder „Beschreiben Sie die Unterschiede zwischen dem ‚walled fortress‘ und ‚defence in depth‘-Ansatz und erläutern Sie jeweils deren Zusammenhang mit Resilienz“. Dabei geht es nicht um die Anwendung von Programmierkenntnissen, sondern primär um die klassifikatorische Einordnung von verschiedenen Cyberaktivitäten, was Aufschluss auf Konfliktdynamiken mit Blick auf Kosten, Komplexität und Invasivität geben soll.

Im Zuge der Pandemie wurden neue Aufgaben entwickelt, die während der Übung in Breakout-Rooms in Gruppen für ca. 60–80 min bearbeitet wurden. Danach stellten zwei Gruppen ihre Ergebnisse vor, welche daraufhin im Plenum diskutiert und ergänzt wurden. Zu Beginn der Übung werden die vergangenen Inhalte mit einem Quiz rekapituliert. Dies dient dazu, vor Ort sowie online die aktive Teilnahme aller und ihre Selbstüberprüfung zu fördern. Gleichzeitig fungiert die Rekapitulation als Erinnerung an den Stand innerhalb der Veranstaltung, was als Überleitung zu den aktuellen Sitzungsthemen genutzt werden kann. Neben der Sicherung des Wissens aus der Vorlesung dient die Übung dazu, die Anwendung auf aktuelle und reale Fälle zu erlernen und Zusammenhänge zu identifizieren. Ein zentrales Lernergebnis ist es, zu identifizieren, wie der Einsatz neuer Technologien Frieden und Konflikte verändert, in welchen Szenarien diese aber auch nur eine Erweiterung bereits lange genutzter Strategien und Entwicklungen darstellen und daher als Mittel für einen politischen Zweck dienen. Ein weiteres Ziel ist, Herausforderungen und Potenziale für friedensfördernde Maßnahmen differenziert zu betrachten, aber auch trotz allen Widrigkeiten konstruktive Lösungsansätze zu entwickeln und anhand historischer Fälle Beispiele für eine gelungene Regulierung und erfolgreiche Vertrauensbildung zwischen Staaten zu vermitteln. Die Auseinandersetzung mit der Rolle der Technologie in der nationalen und internationalen Sicherheitspolitik unterstützt Studierende dabei, ihre Rolle in zukünftigen Tätigkeiten kritisch zu reflektieren. Zudem werden internationale Organisationen und Berufsfelder vorgestellt (z. B. die NGO ICT4PEACE oder die Tätigkeit bei Cyber Emergency Response Teams), die Perspektiven für berufliche Tätigkeitsfelder eröffnen, die sich für Frieden und Sicherheit einsetzen.

Während für die Studierenden der Informatik Konzepte wie hybride Kriege oder klassische IB-Theorien meist komplett neu sind, erlangen die Friedens- und Konfliktforscher*innen neue Einblicke, wenn es zum Beispiel um technische Umsetzung von Angriffen und deren Vereitelung geht. Die Studierenden der verschiedenen Disziplinen wählen häufig die Übungsfragen, die ihren Kenntnissen entsprechen und können somit den Studierenden der anderen Disziplin gegenüber als Expert*innen auftreten. Somit entstehen sowohl ein größeres Verständnis als auch eine größere Wertschätzung der anderen Disziplin.

### Prüfungsleistung

Die Lernziele der Veranstaltung werden über eine Klausur abgeprüft. Dabei liegt der Schwerpunkt darauf, die Inhalte anhand von mehreren fallstudienartigen Aufgaben aus verschiedenen Themenschwerpunkten zu diskutieren und, unter Verwendung von Fachvokabular und mit Referenz auf reale historische Fälle, Abkommen oder technische Methoden, einzuordnen. Dies entspricht auch der Aufgabenstellung der Übung, die damit gezielt auf die Lernziele hinarbeitet und auf die Klausur vorbereitet. Um die aktive Teilnahme während des Semesters zu fördern, gibt es die Möglichkeit, einen Klausurbonus zu erhalten. Dieser wird durch das zweimalige Vorstellen einer Aufgabenlösung im Plenum erreicht. In den Präsenzsemestern wurden die Aufgaben hauptsächlich alleine bearbeitet und vorgestellt, wohingegen die Antworten in den Online-Semestern von der ganzen Gruppe erarbeitet und gemeinsam präsentiert wurden. Die Gruppen wurden zunächst ausgelost, um die aktive Bearbeitung in allen Kleingruppen zu fördern und soziale Interaktion zu ermöglichen. Im Laufe des Semesters wurden dann Gruppen bevorzugt, die noch nicht präsentiert hatten. Die erarbeiteten PowerPoint-Folien wurden von den Dozierenden kommentiert und ebenso online zur Verfügung gestellt. Bonuspunkte können zudem auch erworben werden, indem ein Quiz mit Wiederholungsfragen zu den wichtigsten Inhalten der vorherigen Sitzung erstellt oder eine Präsentation zu einem nicht-klausurrelevanten Thema als Video in Moodle zur Verfügung gestellt wird. Je nach Studiengang konnten in der Lehrveranstaltung 6 (für Informatik-Studiengänge) oder 3 bzw. 8 ECTS (Friedens- und Konfliktforschung, mit oder ohne Modulabschlussprüfung) erworben werden.

## Evaluation und Reflektion

Die Veranstaltung wurde jedes Semester über standardisierte Evaluationsbogen für Lehrveranstaltungen der TU Darmstadt (EvaSys) evaluiert, welche Einblicke in die Perspektive der Studierenden (N_VL_ = 87, N_Ü_ = 98) gibt. Insgesamt wurde die Veranstaltung sehr gut bis gut gesamtheitlich bewertet („Gesamtnote VL“ = 1,67–1,89, Durchschnittsnote Lehrkraft: 1,2–1,47; „Gesamtnote Ü“ = 1,88–2,23). Neben der* „sachlichen Diskussion“*, der „*angenehme[n] und respektvolle[n] Atmosphäre*“ und der Ermutigung zur Beteiligung wird die Wiederholung als positiv erachtet: durch die „*Kombination aus Buch, Vorlesung und Übung […] wiederholt man es automatisch drei Mal und behält es sich sofort*“. Auch der digitale Lehrbetrieb wurde behandelt: „*Vorlesung in digitaler Form ist sehr gelungen, macht Spaß und regt sehr dazu an sich mehr mit dem Thema zu beschäftigen*“ und „*Die vielen Umfragen haben mir sehr gefallen“ *und es sei* „ein leuchtendes Beispiel für den Einsatz digitaler Lehrangebote*“. Die Kombination der verschiedenen Tools wurde ebenfalls hervorgehoben: „*In der Vorlesung wurde verschieden digitale Tools sinnvoll eingesetzt, um die Veranstaltung aufzulockern und die Studierenden einzubeziehen.*“

Einige Antworten adressieren die Interdisziplinarität: Die Inhalte werden* „auch für die, die sonst in ihrem Studiengang keine technischen Hintergründe haben/benötigen*“ als verständlich gestaltet empfunden. In dieser Hinsicht wird das Ziel der Veranstaltung erreicht. Studierende der Informatik merken dagegen oft und über die Jahre hinweg an, dass es die erste Veranstaltung sei, bei der es kein „richtig oder falsch“ gebe. Dies verweist auf einen Lerneffekt, der eine Perspektiverweiterung mit Blick auf unterschiedliche Wissenschafts- und Realitätszugänge umfasst. Gleichzeitig war es bisher meist, aufgrund des Einführungscharakters, nicht gänzlich möglich zu vermitteln, dass es unterschiedliche Verständnisse von Wahrheit auch innerhalb der Geistes- und Sozialwissenschaften gibt sowie der Anspruch einer systematischen Vorgehensweise verschiedenen Disziplinen unabhängig von der konkreten Methode und Forschungsgegenstand gemein sein kann. Zum Teil wurde sich auch ein „*geringerer gesellschaftspolitischer Fokus und höherer technischer Fokus*“ gewünscht, wobei dieser Aspekt sicherlich je nach Studienhintergrund und persönlichem Interesse unterschiedlich ausgeprägt war. Ebenfalls wurde dargestellt, dass es „*nahezu unmöglich [sei] der Vorlesung zu folgen, wenn man nicht das Buch vorher gelesen hat*“, wobei das Buch tatsächlich als Grundlektüre bereitgestellt und vorausgesetzt wurde. Auch der Umgang mit englischer Lektüre ist in der Informatik zum Teil weniger geübt. Vielleicht wird auch deshalb die englische Sprache des Lehrbuchs bei einer auf Deutsch gehaltenen Vorlesung als Veränderungsmöglichkeit benannt. Studierende der Internationalen Studien/Friedens- und Konfliktforschung fanden sich, aufbauend auf dem Konsum regelmäßiger Pflichtlektüre, tendenziell routinierter in die Klausurvorbereitung und der Beantwortung der Essayfragen ein. Gleichzeitig ging es auch um den Abbau von Abwehrhaltungen gegenüber technischen Themen („bitte […] weniger Cyber“). Dem kann häufig mit einer angenehmen Lernatmosphäre entgegengewirkt werden, die es den Studierenden erlaubt Nachfragen zu stellen („hilfsbereit“, „auf Studierende wird eingegangen“).

Freitextantworten geben Aufschluss über die studentischen Motive zur Teilnahme an der Veranstaltung. So betont eine Person: „*Die Thematik der Vorlesung ist sehr wichtig und kommt ansonsten im Informatikstudium leider viel zu kurz*“. Interesse an der Auseinandersetzung mit politischen Verhältnissen zeigte sich in der zuletzt digital durchgeführten Reflexionsrunde vor allem mit Blick auf die militärische Nutzung von IT sowie *cyber espionage* im Kontext internationaler Beziehungen. Zudem zeigten sich Informatikstudierende interessiert an der Aktualisierung theoretischer Konzepte mit Blick auf empirische Referenzobjekte im Cyberspace. Das Bewusstsein über aktuelle gesellschaftliche Konflikte konnte ebenfalls geschärft werden, was die Studierende auch in ihrer Rolle als angehende Informatiker*innen für wichtig erachteten.

## Fazit: Kernbeobachtungen

Abschließend und basierend auf vier Iterationen der Durchführung inklusive der Evaluationen lassen sich vier Kernbeobachtungen festhalten. Fokussiert auf eine problem-lösungsorientierte Wissenschaft erkennen wir erstens eine hohe empirische Relevanz einer Auseinandersetzung aus Perspektive der naturwissenschaftlich-technischen Friedens- und Konfliktforschung, welche sich auch in der universitären Lehre niederschlägt (siehe (1) Friedens- und Sicherheitspolitische Notwendigkeit). Die systematische Bearbeitung der Themenkomplexe betreffend, zeigt sich, dass die Lehrveranstaltung Limitationen der Wissenschaftsumgebung aufzeigt und versucht, in diesem Kontext Potenziale für die Forschung zu aufzugreifen (siehe (2) Disziplinäre Grenzen der Natur‑, Ingenieur- oder Sozialwissenschaften, (3) Komplementärer Wissens- und Kompetenzerwerb). Daran anschließend stellt sich die Frage nach dem substanziellen Zugewinn, welcher sich durch die interdisziplinäre Herangehensweise auch mit Blick auf sich verändernde Phänomene der Realwelt ergibt (siehe (4) Konzept-Transfer und nachhaltige Anwendbarkeit).

1. *Friedens- und Sicherheitspolitische Notwendigkeit*: Ereignisse, wie der Überfall auf die Ukraine 2022, machen die Bedeutung fundierter Kenntnisse der Friedens- und Konfliktforschung als Ganzes, aber speziell auch der naturwissenschaftlich-technischen Friedensforschung mit Bezügen zur Physik, Biologie, Chemie, Informatik, Elektrotechnik, Maschinenbau und anderen technischen Disziplinen für die kritische Einordnung von Technologien in Konflikten deutlich und rücken die dringende Empfehlung des Wissenschaftsrats (Wissenschaftsrat 2019) zur Stärkung dieses Bereichs in ein noch grelleres Licht. Für angehende Friedens- und Konfliktforscher*innen erlaubt eine solche Lehrveranstaltung ein Anknüpfen an relevante Themenkomplexe, wovon auch theoretische Debatten mit Blick auf ihre Strahlkraft profitieren. Für Informatikstudierende, die sich zukünftig in einer stark gestalterischen Rolle einfinden werden, ist die Lehrveranstaltung ein sinnvoller Raum für Reflexion über die gesellschaftlichen Auswirkungen von IT, was auch in das Design von Artefakten einfließen kann. Insbesondere das Feedback aus technischen Studienrichtungen verdeutlicht, dass hier nach wie vor ein hoher Aufklärungs- und Mobilisierungsbedarf bei Studierenden besteht, Technik als Bestandteil von Gesellschaften und Gestalter von gesellschaftlichen Prozessen zu verstehen. Die sozialen, ethischen und in diesem Fall sicherheitspolitischen Konsequenzen technischer Produkte und Verfahren und die eigene Beteiligung daran zu reflektieren ist eine weiterhin dringende Notwendigkeit im Bereich der technischen Studienrichtungen. Gleichzeitig haben die Diskussionen mit Studierenden aber auch gezeigt, dass der Ansatz, neben der eigenen Verantwortung auch Gestaltungsmöglichkeiten mit Hilfe technischer Fähigkeiten zu vermitteln, sehr dankbar und oft mit großer Begeisterung angenommen wird.
2. *Disziplinäre Grenzen der Natur‑, Ingenieur- oder Sozialwissenschaften: *Bei einer von der Thematik und Hörer*innenschaft interdisziplinären Veranstaltung besteht immer das Risiko, dass diese je nach Hintergrund als zu technisch oder zu gesellschaftswissenschaftlich eingeschätzt wird. Als sehr wichtig haben wir hier empfunden, mögliche Hürden oder Hemmnisse zum Besuch dieser Veranstaltung abzubauen (vgl. „Ich habe keinerlei Informatik-Kenntnisse, kann ich dennoch teilnehmen?“) und alle dazu zu ermuntern, ihren Beitrag zu leisten. Überdies besteht in darauf aufbauenden Seminaren, Forschungspraktika oder Abschlussarbeiten die Möglichkeit der zielgruppengerechten Vertiefung. Die Integration bekannter Praktiken aus den jeweils involvierten Studiengängen erlaubt Orientierungspunkte, die die thematische Auseinandersetzung erleichtern. Ebenso erleichtern die vielfältigen Lehrformate (Vorlesung, Übung, Buch, E‑Learning) den Einstieg für Studierende mit unterschiedlichen Routinen und Lernkompetenzen. Die Verstetigung sozialer Interaktion auch in Zeiten digitaler Lehre ist unabdingbar für einen inhaltlichen Austausch über Themen, bei denen sich nicht alle immer „Zuhause“ fühlen, wobei ein iterativer Charakter der Lehrveranstaltung zusätzliche Sicherheit bieten kann. Da innerhalb der jeweiligen Disziplinen eine Spezialisierung auf bestimmte Bereiche im Zuge des Studiums erfolgt, fällt die fehlende Fokussierung auf Auseinandersetzungen mit Themengebieten, die disziplinäres Hintergrundwissen erfordern, nicht immer positiv auf. In interdisziplinären Lehrveranstaltungen ist es daher wichtig, sich sinnvoll an den verschiedenen Wissenschaftskulturen zu bedienen sowie für eine offene Fehlerkultur zu werben, um Dialog anzustoßen.
3. *Komplementärer Wissens- und Kompetenzerwerb: *Es ist relativ wahrscheinlich, dass Studierende unterschiedlicher Studiengänge andere Dinge erlernen müssen. Während für die einen DDoS-Angriffe, Verschlüsselungsalgorithmen, Vulnerabilitäten, Exploits und Backdoors technisch bereits voll verständlich sind, haben andere bereits beste Kenntnisse der Konzepte positiver und negativer Frieden, Versicherheitlichung, oder der Mechanismen der Rüstungskontrolle oder Verifikation. Durch ausgewogene Fragen erhält mal die eine, mal die andere Disziplin die Gelegenheit, als Expert*innen aufzutreten. Gleichzeitig müssen auch Unterschiede der Disziplinen adressiert werden: Das argumentative Abfragen von Wissen muss bereits in einem geeigneten Lehrformat wie der Übung vermittelt werden, damit in einer Klausur, die entsprechendes Können abfragt, gleiche Chancen der erfolgreichen Bearbeitung bestehen. Im Vorgang der Prüfung werden die unterschiedlichen Hintergründe im Sinne eines komplementären Lernens nutzbar gemacht, während in der Prüfung Spezialisierungen keine Rolle mehr spielen, sondern es um den Transfer eines allgemein zugänglich gemachten Wissenstandes auf Problemszenarien geht.
4. *Konzept-Transfer und nachhaltige Anwendbarkeit: *Ergänzend zum bereits existierenden Lehrbuch zur naturwissenschaftlich-technischen Friedensforschung als Ganzes (Altmann et al. 2017), wurde die Übertragbarkeit der Konzepte u. a. durch das Lehrbuch *Information Technology for Peace and Security* (Reuter 2019) aktiv gefördert. Anzumerken ist hier, dass aufgrund der Dynamik des technischen Entwicklungsstandes weitere Auflagen notwendig werden. Dies ist der Fall, da andernfalls dargestelltes Wissen und Einordnungen schnell als veraltet gelten können. Gleichzeitig geht es einer interdisziplinären Fokussierung auf IT und Frieden nicht um die Aneinanderreihung diverser, teilweise unzusammenhängender Themen. Vielmehr erweisen sich Zugänge als nachhaltig, die sich auf einer „mittleren“ Ebene der Abstraktion ansiedeln. So wenig wie Informatik-Studierende Meta-Theorien oder Großtheorien der IB für sich nutzbar machen können, so wenig bringt spezialisiertes technisches Wissen Studierende der Friedens- und Konfliktforschung in diesem Rahmen voran. Auch erlaubt eine Anwendung theoretischer Konzepte auf Fallbeispiele oder ein Rückgriff auf „Theorien mittlerer Reichweite“ das Zusammentreffen unterschiedlicher epistemologischer Hintergründe, wodurch die interdisziplinäre Debatte nachhaltig gestärkt werden kann. Eine wichtige Grundlage des Lehrbuches und der Vorlesung besteht in der interdisziplinären und ausgeprägt heterogenen Gruppe der Autor*innen des Lehrbuches und der Ausgestaltung der Lehrenden. Diese ermöglicht es, sowohl Domänen-Wissen vertieft zu vermitteln, dieses gleichzeitig aber auch in der Breite zu verankern und in andere Kontexte zu setzen. Eine solche Vermittlung wäre aber bspw. auch durch unkonventionellere Lehrformate, wie Ringvorlesungen und Übungen mit Gast-Lehrenden aus unterschiedlichen Fachrichtungen oder mit Praxisbezug zu Friedens- und Sicherheitsfragen denkbar. Insbesondere mit Blick auf die technischen Fragen und Grundlagen erscheint es uns wesentlich, die technischen Zusammenhänge auch in sozialwissenschaftlich geprägten Diskursen mitzudenken und ggf. durch externes *Know-How* einzubeziehen. Lehrveranstaltungen, die auf einem solchen Lehrbuch aufbauen und zusätzliche Expert*innen zu aktuellen Thematiken einschließen, können auch von Lehrstühlen-/personen durchgeführt werden, welche zuvorderst innerhalb ihrer disziplinären Grenzen arbeiten.
